# Noncholesteatomatous Cyst of the Tympanic Membrane: A Nonpublished Entity?

**DOI:** 10.1155/2015/187187

**Published:** 2015-09-09

**Authors:** Rafael Ramírez-Camacho, Isabel Salas, Almudena Trinidad, Ithzel Maria Villarreal

**Affiliations:** ^1^Otorhinolaryngology Department, “Puerta de Hierro” University Hospital, “Universidad Autónoma de Madrid”, 28222 Madrid, Spain; ^2^Pathology Department, “Puerta de Hierro” University Hospital, “Universidad Autónoma de Madrid”, 28222 Madrid, Spain

## Abstract

*Introduction*. The presence of a serous cyst in the tympanic membrane implies the description of a new or unpublished entity based on our knowledge whose origin may be very unlikely explained on actual embryologic and anatomic background. *Clinical Case*. We present a case of a 45-year-old woman with progressing right hearing loss. Physical examination revealed a whitish, round-shaped malformation in the posterior-inferior quadrant of the right tympanic membrane. The cyst was removed with a transcanal tympanoplasty. *Discussion*. A thorough PubMed search that involved the terms tympanic membrane gland, epithelial inclusion cysts, mucous-secreting cyst, and tympanic cyst has shown no positive results. The first description of an unknown entity, such as a tympanic membrane serous cyst, may be the key for clinicians to start paying attention to patients who suffer from similar pathologies and may pass unnoticed because of their rarity or peculiarity.

## 1. Introduction 

Most of the cysts described in the middle ear are of cholesteatomatous nature and are related with infectious history or iatrogenic effects [1, 2]. Occasionally, malformations associated with the first pharyngeal pouch have been described in the external auditory canal situated lateral to the tympanic membrane [3].

The presence of a serous cyst situated in the tympanic membrane implies the description of a new or unpublished entity based on our knowledge whose origin may be very unlikely explained on actual embryologic and anatomic background.

## 2. Clinical Case

We present a case of a 45-year-old woman who assisted our ENT consult complaining of a slowly progressive right hearing loss. She denies any traumatic event, infections, or surgery in that ear.

**Figure 1:**
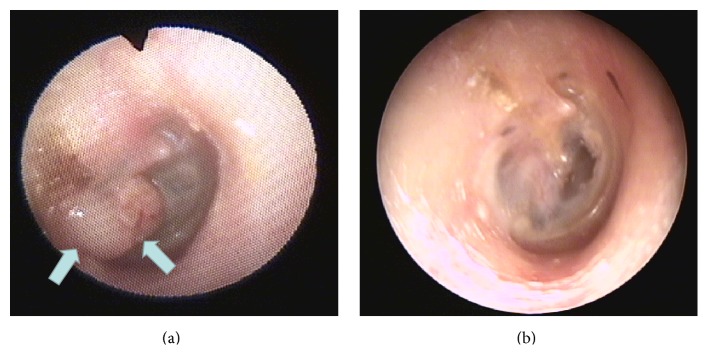
Preoperatory (a) and postmyringoplasty (b) otomicroscopic images.

Physical examination revealed a whitish, round-shaped malformation located in the posterior-inferior quadrant of the right tympanic membrane (Figure 1). The audiometric results showed a conductive hearing loss with a 30 dB gap in all frequencies. A transcanal tympanoplasty was performed. After the removal of the cystic lesion the residual membrane perforation was restored with an underlay myringoplasty technique with posterior tragal perichondrium. The postoperative audiometric evaluation showed that the previous 30 dB gap found in all frequencies was corrected.

**Figure 2:**
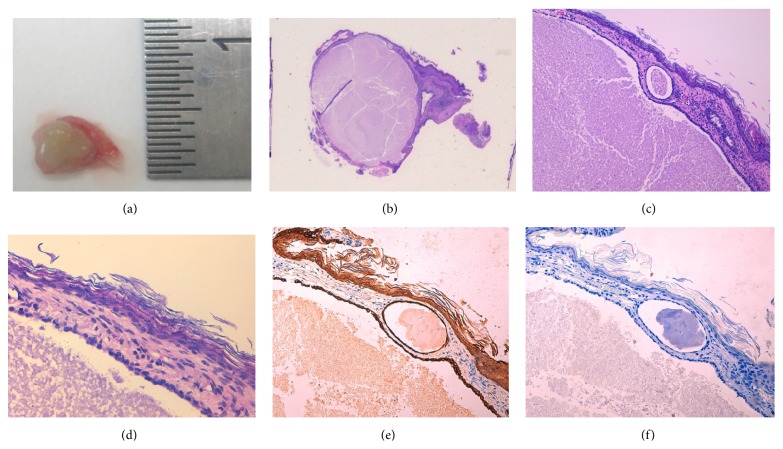
A 0.5 cm yellowish nodule (a). Histological macroview (HE 1.5x) (b). Multiloculated cystic formation lined by a monostratified nonmucin productor cuboidal epithelium (400x HE and 630x HE) (c) and (d). Immunohistochemical analysis shows cytokeratin-positive cells (AE1 and AE3) (e) and negativity for S100 protein, neural specific enolase, glial fibrillary acidic protein (GFAP), and synaptophysin (400x) (f).

Histopathological results showed the existence of a multilocular cystic malformation with an internal serous component. No cholesteatomatous component was observed and well defined margins were distinguished (Figure 2).

## 3. Discussion

The tympanic membrane is the result of the final invagination of the first pharyngeal arch which permeates to the mesoderm of the first pharyngeal cleft until it contacts the endoderm of the first pharyngeal pouch leaving between them a thin mesodermal layer. In adults, the result of this fusion is present in the 3 layers that form the tympanic membrane, which separates the external acoustic canal from the middle ear and measures approximately 1 cm in diameter. The tympanic membrane is quite thin despite its double layering and it really is a dividing element designed by epithelial cells. The outer concave layer is outlined by stratified squamous epithelium, while the inner convex layer is composed by low columnar epithelium. A firm connective tissue layer rests in between both of them.

Presumably, there is no glandular component with the possibility of forming a cyst in the tympanic membrane. Ceruminous glands in the external ear canal skin are found far distant from the membrane and their morphologic characteristics are different from the ones found in our patient. Ceruminous gland tumors, in their benign forms, are settled in the cartilaginous portion of the external acoustic canal [3].

A thorough PubMed search that involved the terms tympanic membrane gland, epithelial inclusion cysts, mucous-secreting cyst, and tympanic cyst has shown no positive results. The only reference found with a similar description shows a patient with a facial palsy and a histologically similar cyst but situated in the posterior-inferior portion of the internal tympanic cavity not including the tympanic membrane. The authors believe this case was originated in the first pharyngeal pouch [4]. Another case involving a child describes a branchial cleft anomaly with two fistulous tracts, one of which was associated with an unusual otoscopic finding. A type II first branchial cleft cyst after an acute infection is described showing in the otomicroscopy a fibrous band arising from the wall of the canal and attached to the tympanic membrane at the umbo. Some similarities with our case are observed but the location and the fibrous component of the cyst differ [5].

The presence of epidermal/epithelial inclusion cysts in the tympanic membrane has only been described as a possible iatrogenic lesion occurring after a myringoplasty or after infection [1, 6, 7]. The lack of infectious, traumatic, or surgical history in our patient excludes the possibility of an exogenous origin.

Most congenital epidermoid cysts arise due to an embryologic accident during the 3rd and the 5th weeks of gestation; they may result from abnormal sequestration or invagination of the ectoderm along the sites of dermal fusion [1, 8]. Nevertheless, the tympanic membrane lacks these coalescent sites. Histologically, the lesion described in our clinical case is similar to the tendinous ganglion cysts because of their mesodermal root. Ganglion cysts arise from a myxoid degeneration of connective tissue of the joint capsule secondary to trauma.

The exclusive removal of the cyst from the tympanic membrane is certainly a surgical challenge enclosing the risk of leaving an area with less resistance and thus susceptible to secondary perforation. The complete resection of the cyst including the epithelial and endothelial layers usually results in a precise well vascularized perforation. A myringoplasty is a procedure which deals with repairing the perforated membrane and may be done with a postaural, endaural, or endomeatal approach [9]. Depending on the placement of the graft material used the technique may be classified as underlay technique, overlay technique, interlay technique, or its combinations. The graft material in the underlay technique is placed under the membrane remnant including the flap after elevating the tympanomeatal flap [9]. We have obtained promising and satisfactory results applying this technique as we did in our case.

Each pathological entity in a human being may have an equivalent or identical case which may affect other people. The opposite situation would be the first description of a particular case. Even though this may literally be correct, practical reasoning discards this possibility. An entity may have always existed but never been described before.

Presenting clinical cases has a low value within scientific literature. However, the first description of an unknown entity may be the key for clinicians to start paying attention to patients who suffer from similar pathologies that may pass unnoticed because of their rarity or peculiarity.

## Summary

The following are some aspects of noncholesteatomatous cyst of the tympanic membrane:

- It is the first description of an unknown entity.
- It passes unnoticed because of its rarity or peculiarity.
- It is difficult to be justified embryologically.
- There is lack of bibliographic reference data at the moment.
- There is lack of infectious, traumatic, or surgical history.
- There are similar descriptions but none with the same location.
- Exclusive removal involves a surgical challenge.
